# Fractalkine (CX3CL1) and Its Receptor CX3CR1: A Promising Therapeutic Target in Chronic Kidney Disease?

**DOI:** 10.3389/fimmu.2021.664202

**Published:** 2021-06-07

**Authors:** Sarah Cormican, Matthew D. Griffin

**Affiliations:** ^1^ Regenerative Medical Institute (REMEDI) at CÚRAM Centre for Research in Medical Devices, School of Medicine, College of Medicine, Nursing and Health Sciences, National University of Ireland, Galway, Ireland; ^2^ Nephrology Services, Galway University Hospitals, Saolta University Health Group, Galway, Ireland

**Keywords:** CX3CL1 (fractalkine), CX3CR1, chronic kidney disease, fibrosis, macrophage, innate immunity, renal fibrosis, renal inflammation and fibrosis

## Abstract

Innate immune cells are key contributors to kidney inflammation and fibrosis. Infiltration of the renal parenchyma by innate immune cells is governed by multiple signalling pathways. Since the discovery of the chemokine fractalkine (CX3CL1) and its receptor, CX3CR1 over twenty years ago, a wealth of evidence has emerged linking CX3CL1-CX3CR1 signalling to renal pathologies in both acute and chronic kidney diseases (CKD). However, despite the extent of data indicating a pathogenic role for this pathway in kidney disease and its complications, no human trials of targeted therapeutic agents have been reported. Although acute autoimmune kidney disease is often successfully treated with immunomodulatory medications, there is a notable lack of treatment options for patients with progressive fibrotic CKD. In this article we revisit the CX3CL1-CX3CR1 axis and its functional roles. Furthermore we review the accumulating evidence that CX3CL1-CX3CR1 interactions mediate important events in the intra-renal pathophysiology of CKD progression, particularly *via* recruitment of innate immune cells into the kidney. We also consider the role that systemic activation of the CX3CL1-CX3CR1 axis in renal disease contributes to CKD-associated cardiovascular disease. Based on this evidence, we highlight the potential for therapies targeting CX3CL1 or CX3CR1 to benefit people living with CKD.

## Introduction

Innate immune cells are key contributors to kidney inflammation and fibrosis. Infiltration of the renal parenchyma by innate immune cells is governed by multiple signalling pathways. Since the discovery of the chemokine fractalkine (CX3CL1) and its receptor, CX3CR1 over twenty years ago, a wealth of evidence has emerged linking CX3CL1-CX3CR1 signalling to renal pathologies in both acute and chronic kidney diseases (CKD). However, despite the extent of data indicating a pathogenic role for this pathway in kidney disease and its complications, no human trials of targeted therapeutic agents have been reported. Although acute autoimmune kidney disease is often successfully treated with immunomodulatory medications, there is a notable lack of treatment options for patients with progressive fibrotic CKD. In this article we revisit the CX3CL1-CX3CR1 axis and its functional roles. Furthermore we review the accumulating evidence that CX3CL1-CX3CR1 interactions mediate important events in the intra-renal pathophysiology of CKD progression, particularly *via* recruitment of innate immune cells into the kidney. We also consider the role that systemic activation of the CX3CL1-CX3CR1 axis in renal disease contributes to CKD-associated cardiovascular disease. Based on this evidence, we highlight the potential for therapies targeting CX3CL1 or CX3CR1 to benefit people living with CKD.

## Discovery of the CX3CL1-CX3CR1 Axis as a Regulator of Chemotaxis and Adhesion

CX3CL1 was identified independently by two separate research groups in 1997 and termed neurotactin and fractalkine by the respective authors (1, 2). Neurotactin may also refer to a surface glycoprotein first identified in *Drosophilia melanogaster* and this term has therefore not remained in extensive use (3, 4). Throughout this article we will refer to this unique chemokine as CX3CL1.

Pan et al. identified CX3CL1 following sequencing of the murine choroid plexus while Bazan et al. identified it by searching for chemokine-like sequences in an expressed sequence tag database in the National Centre for Biotechnology Information (NCBI). Both groups identified a cDNA clone which corresponded to a large protein containing a sequence of hydrophobic residues suggestive of a transmembrane domain. Pan et al. reported that messenger RNA for CX3CL1 was identified in most human tissues with the notable exception of peripheral blood cells but corresponding analyses of protein expression were not reported in this paper. Human endothelial and epithelial cell lines transfected with the coding region of CX3CL1 were found to express CX3CL1 on the cell surface, consistent the hypothesis that the newly discovered protein was membrane-bound. Bazan et al. showed that CX3CL1 could also be released into cell culture supernatants. The authors described disparate results for chemotactic assays with CX3CL1 which was reported to induce *in vitro* neutrophil chemotaxis by Pan et al. and *in vitro* monocyte and lymphocyte chemotaxis by Bazan et al. The latter also reported that surface CX3CL1 triggered adhesion of monocytes or T-cells. Notably, Pan et al. observed that CX3CL1 was upregulated in the brains of mice treated with lipopolysaccharide or in mice with experimental autoimmune encephalitis, suggesting a pro-inflammatory role. Thus, CX3CL1 was demonstrated to be a new and structurally unique chemokine which was comprised of CX3C domain and a mucin stalk. The CX3C domain may be shed from the mucin stalk to act as a soluble chemokine or may be expressed on the cell surface *via* this transmembrane mucin stalk.

In the same year, Imai et al. identified that the orphan receptor V28 was a high-affinity receptor for CX3CL1 and that this receptor mediated *in vitro* CX3CL1-induced chemotaxis (5). This G protein coupled receptor (GPCR) was previously reported by Raport et al. who used degenerate PCR to identify GPCRs related to the IL-8 receptor with potential relevance to immune function. Notably V28 had been identified as highly expressed in neural and lymphoid tissue but no known chemokines were found to trigger Ca^2+^ influx in V28 transfected cell lines (6). In contrast, CX3CL1 ligation of the V28 receptor induced calcium influx which was inhibited by pertussis toxin, indicating that CX3CL1 signal transduction is mediated by a Gαi class G-protein. This work further demonstrated that V28 is capable of inducing cellular adhesion through a mechanism independent of G-protein signalling. V28 surface expression was identified on natural killer cells, T-cells and monocytes but not on granulocytes and was capable of triggering trans-endothelial migration of both lymphocytes and monocytes. Finally, endothelial expression of the fractalkine protein was shown to induce adhesion of V28 transfected cells or natural killer cells to the endothelium (5). The mechanism of adhesion was shown to require both the CX3C and mucin stalk domains of the CX3CL1 protein. Overall, these initial studies demonstrated that CX3CL1 functions as both a chemokine and an adhesion molecule *via* binding of the V28 receptor. The V28 receptor was subsequently re-named CX3CR1 and remains the only receptor through which CX3CL1 has been shown to function (5). Combadiere et al. reached similar conclusions and also reported that CX3CR1 may function as a fusion co-receptor in HIV infection (7).

In the years following these discoveries, the role of the CX3CL1-CX3CR1 axis in inducing chemotaxis and adhesion of leucocyte populations was pursued in detail by several groups. Al-Aoukaty et al. further confirmed that engagement of CX3CR1 results in G-protein activation in the cellular membrane, specifically G_i_ or G_z_ proteins (8). Al Aoukaty et al. postulated that the mucin stalk contained within CX3CL1 was key to the mechanism of CX3CL1-CX3CR1 mediated adhesion. CX3CL1-CX3CR1 interactions were shown by Fong et al. to be capable of inducing monocyte, CD8^+^ lymphocyte and NK-cell arrest and firm binding under physiologic flow conditions, including when CX3CL1 was expressed on the surface of endothelial cells (9). Work by Goda et al. indicated that exposure to CX3CL1 also enhances the ability of both THP-1 cells and *ex vivo* monocytes to bind to integrins and fibronectin (10). With regards to the effect of inflammation on regulation of CX3CL1-CX3CR1 signalling, Harrison et al. demonstrated that CX3CL1 is expressed at a low level by rat endothelial cells in steady state but that both mRNA and protein expression are increased after *in vivo* treatment with lipopolysaccharide (LPS) or the pro-inflammatory cytokines tumour necrosis factor-alpha (TNF-α) and interleukin-1 (IL-1) (11). It was subsequently demonstrated that CX3CL1 induction by pro-inflammatory stimuli is mediated by nuclear factor kappa-light-chain-enhancer of activated B cells (NF-κB) signalling (12).

In relation to the kidney, the signalling pathways involved in TNF-α−mediated CX3CL1 expression within mesangial cells were first dissected by Chen et al. (13). Mesangial cells stimulated with TNF-α upregulate CX3CL1 expression at the mRNA and protein levels. The protein produced is cleaved by matrix metalloproteinases (MMPs) and can then induce transmigration of a monocytic cell line. Intracellular signalling cascades triggered by TNF-α were investigated using protein kinase inhibitors and inhibitors of NF-κB. Inhibitors of protein kinase C (PKC) or of p42/44 (ERK1/2) mitogen activated protein kinase (MAPK) reduced CX3CL1 expression after TNF-α stimulation, as did inhibition of NF-κB or activation protein-1 (AP-1). Overall, these data indicate that TNF-α induces CX3CL1 through multiple intracellular signalling including PKC, ERK1/2 MAPK, NF-κB and AP-1.

## Physiological Roles of CX3CL1-CX3CR1 Beyond Chemotaxis

A further physiological role for CX3CR1 was reported by Auffray et al. (14). This work used real-time intra-vital microscopy and CX3CR1^gfp/+^ mice to demonstrate that Ly6C^lo^ monocytes crawl on the luminal surface of blood vessels and extensively patrol the endothelial surface (14). This patrolling process, which is dependent on both the integrin LFA-1 (CD11a/CD18) and CX3CR1, plays a role in immune surveillance and allows early response to tissue damage or infection. Subsequently, Cros et al. demonstrated, using adoptive transfer of human nonclassical monocytes into *Rag* ^-/-^ mice, that human nonclassical monocytes, the equivalent of murine Ly6C^lo^ monocytes, also patrol the vasculature (15).

CX3CL1-CX3CR1 signalling has also been reported to play an anti-apoptotic role in myeloid cell development and survival during steady-state conditions (16). Lyszkiewcz et al. demonstrated that, in steady-state, CX3CR1 deficiency results in accelerated turnover of myeloid precursors in the bone marrow. In a competitive adoptive transfer model, CX3CR1-deficient dendritic cells, monocytes, macrophages and granulocytes have a survival disadvantage. These effects are not seen in animals subjected to sub-lethal whole body irradiation suggesting that the survival disadvantage conferred by CX3CR1 deficiency is overcome in inflammatory conditions (17). This indicates a role for the CX3CL1-CX3CR1 pathway in homeostatic but not inflammation-driven generation of myeloid cells. It has also been demonstrated, however, that anti-apoptotic signalling through the CX3CL1-CX3CR1 pathway is important for monocyte survival within atherosclerotic plaques (18). This elegant work demonstrated less severe atherosclerosis with reduced monocyte survival and increased apoptotic monocytes within atherosclerotic plaques of CX3CR1-deficient mice - effects that were reversed by enforced expression of the anti-apoptotic protein Bcl-2. Furthermore, cultured human monocytes are rescued from cell death by treatment with recombinant CX3CL1. The anti-apoptotic effects of CX3CL1 appear to be G-protein mediated as they are abolished by exposure to pertussis toxin. Interestingly, monocytes isolated from individuals carrying the M280 mutation in the CX3CR1 gene are not rescued from apoptosis by exposure to CX3CL1 (19) and homozygotes for this mutation are protected from atherosclerotic cardiovascular disease (20) while being at increased risk of systemic candidiasis (21) – suggesting important, non-redundant, biological roles for this pathway in human atherogenesis and immunity. CX3CL1-CX3CR1 anti-apoptotic signalling may also enable T-cell survival during inflammation (22). Furthermore, CX3CL1 mediates survival and proliferation of vascular smooth muscle cells through ERK- and Akt-dependent signalling - with Akt phosphorylation mediated by transactivation of the epidermal growth factor receptor (EGFR) (23).

**Figure 1:**
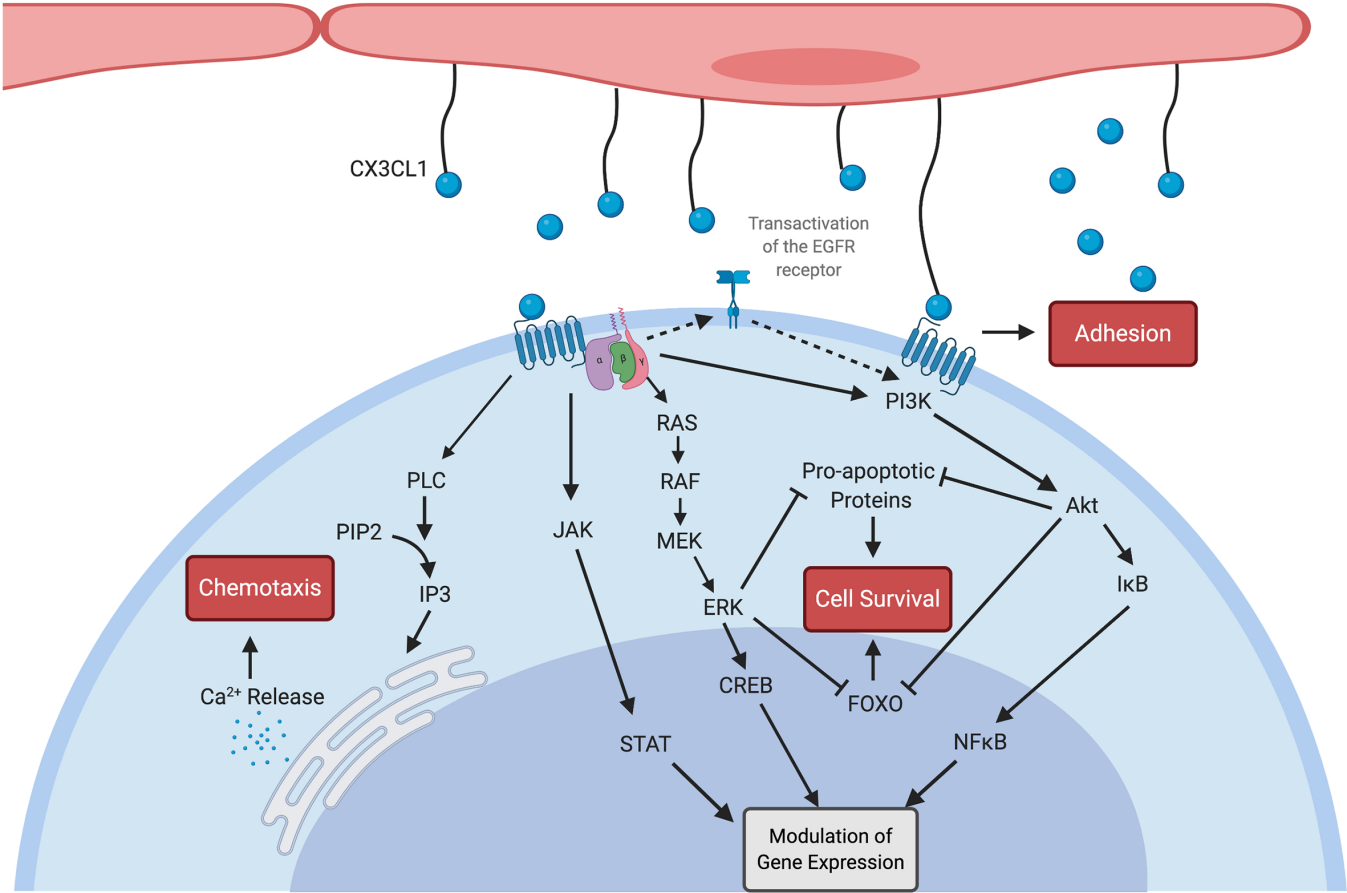
Multiple signalling pathways are activated by both soluble and membrane-bound CX3CL1 (blue spheres) as outlined here. CX3CR1 is a G-protein coupled receptor linked to Gi and Gz proteins (8). Calcium release resulting in chemotaxis was demonstrated by Harrison et al. (29). CX3CL1 also contributes to intercellular adhesion through mechanisms which do not appear to be G-protein sensitive (5). Enhanced cellular survival is mediated by both the Akt and ERK pathways, including *via* transactivation of the EGFR receptor (16). CX3CL1 ligation of the CX3CR1 receptor also leads to nuclear translocation of transcription factors (30) including NFκB (31), CREB (32) and STAT (33) with resulting modulation of gene expression. Created with BioRender.com.

It is evident that the CX3CL1-CX3CR1 axis is key in leucocyte trafficking throughout the body and immune cell survival in certain circumstances. CX3CR1 is also expressed by microglia in the brain and CX3CL1-CX3CR1 is of importance in neurodevelopment and inflammatory disease of the central nervous system (24, 25). Certain cells of mesenchymal origin including smooth muscle cells and mesangial cells also express CX3CR1, as discussed later in this review. Within the central nervous system it has been shown that CX3CL1 signalling may stimulate increased pro-inflammatory cytokine production through in a p38-MAPK dependent fashion (26). Whether such a role exists in other organs or circulating immune cells remains to be seen. CX3CL1- CX3CR1 signalling may be of broader importance in embryonic development, including nephrogenesis, as both CX3CL1 and CX3CR1 are expressed by various structures and cells within the developing kidney (27). **Table 1** summarises the expression of CX3CR1 by different immune cells and its functional roles in these cells. These data present a breakdown of CX3CR1 expression and responses to CX3CL1 by cell type based on data published early in the twenty first century. It should be noted that each leucocyte population referred to in this table is comprised of multiple, functionally distinct, subpopulations. Further work examining lymphocyte and monocyte subtype-specific expression and functional responses would be of value in elucidating the precise role of this pathway in immune cell function. Multiple signalling pathways mediate various cellular responses to CX3CL1 binding to CX3CR1 including intracellular calcium release and the MAP/ERK, PI3K/Akt, JAK/STAT pathways, as illustrated in **Figure 1**.

**Table 1:** Summary of expression of CX3CR1 and chemotactic, adhesive and other physiological responses to CX3CL1 in major immune cell subtypes.

Cell Type	CX3CR1 Expression	Chemotactic Response to CX3CL1	Adhesion Response to CX3CL1	Other Responses to CX3CL1
Monocytes	Yes (higher on non-classical monocytes)	Yes (variable data regarding subset-specific response)	Yes	Crawling on vasculature (nonclassical subset) Survival *via* anti-apoptotic signalling in both steady state and atherosclerosis
Macrophages	Yes	Yes	Not reported	Maturation and survival
Dendritic Cells	High expression by cDC, low or absent expression on pDC	Yes (cDC)	Not reported	Maturation and survival
T-cells	Some subtypes	Some subtypes	Some subtypes	Cell survival in inflammatory lung disease
B-cells	No	No	No	N/A
NK-cells	Yes	Yes	Yes (after activation)	None reported
Neutrophils	No (mRNA expression reported)	Varying Reports*	No	Possible cell survival role in steady-state

cDC, conventional dendritic cells; pDC, plasmacytoid dendritic cells; *Pan et al. reported an effect on neutrophil chemotaxis but this was not shown in subsequent studies, Becker et al. suggested a possible role in in vivo recruitment (28).

## The CX3CL1-CX3CR1 Axis in Kidney Disease: Early Discoveries (1998–2002)

Shortly after the discovery of CX3CL1 and its cognate receptor CX3CR1, studies examining the role of the CX3CL1-CX3CR1 axis in multiple disease states and the potential for therapeutic blockade emerged. The first such study of relevance to kidney disease examined the potential for chemokine receptor blockade by viral macrophage inflammatory protein-II (vMIP-II), a chemokine analogue produced by human herpes virus-8, in experimental anti-GBM glomerulonephritis in Wistar-Kyoto rats (34). This study confirmed that v-MIP-II competitively inhibits CX3CL1 binding to immune cells and showed that administration of v-MIP-II in this rat model of glomerulonephritis attenuated disease severity. However, v-MIP-II is an antagonist of multiple chemokine receptors and there results could not be definitively ascribed to blockade of CX3CL1/CX3CR1 interaction (34). A subsequent study looked more specifically at the effects of CX3CR1 blockade in the same model of acute glomerular inflammation. Expression of both CX3CL1 and CX3CR1 were increased after the induction of anti-GBM glomerulonephritis. CX3CL1 expression was induced on the glomerular endothelium while CX3CR1 expression was identified on infiltrating T-cells and macrophages. Notably, these cells, once isolated from inflamed kidneys, were capable of undergoing chemotaxis towards a CX3CL1 gradient. In keeping with the proposed pathological relevance of CX3CL1 in kidney inflammation, daily treatment with an anti-CX3CR1 antibody resulted in attenuated disease severity (35). In a study of human renal biopsy samples from 23 patients with crescentic glomerulonephritis, CX3CL1 expression was shown to be increased in acute disease and decreased after steroid treatment (36). These findings provoked interest in the therapeutic use of chemokine blockade in renal disease (37, 38).

In 2002, Cockwell et al. examined the expression of CX3CL1 in renal biopsies from patients with acute glomerulonephritis due to ANCA-associated vasculitis (AAV) or with acute transplant rejection, using either normal tissue obtained at the time of tumour nephrectomy or biopsies showing minimal change disease as negative controls. At gene expression level, mRNA for CX3CL1 was increased in the glomeruli of patients with acute AAV and increased to a lesser extent in patients with acute transplant rejection. In the tubulointerstitium, mRNA for CX3CL1 was increased in both groups compared to control tissue but to a greater degree in transplant rejection. At protein level, CX3CL1 expression, determined by immunohistochemistry, was higher in biopsies with renal inflammation. Serial section analysis demonstrated that CX3CL1 positivity was localised to areas of T-cell or macrophage infiltrates (39). Related *in vitro* work demonstrated that proximal tubular epithelial cells (PTEC) upregulate CX3CL1 expression after TNF-α stimulation and that this upregulation is capable of supporting adhesion of either THP-1 cells or *ex vivo* natural killer (NK) cells (40).

Segerer et al. further studied the expression of CX3CR1 by infiltrating cells in human kidney disease using similar control samples (41). Serial section analysis was used to correlate the location of CX3CR1 positivity with the presence of T-cell or monocyte/macrophage infiltrates, identified by anti-CD3 and anti-CD68 staining, respectively. The negative control or uninflamed tissue showed occasional CX3CR1 positive cells and minimal cellular infiltration. By comparison, kidney biopsies of membranous nephropathy, focal segmental glomerulosclerosis or collapsing glomerulopathy had more CX3CR1 positive cells, corresponding to the locations of T-cell or monocyte/macrophage infiltration. In crescentic glomerulonephritis, cellular infiltration was much more pronounced with a preponderance of CD68^+^ monocyte/macrophages. CX3CR1 was highly expressed and, similarly, corresponded to the location of monocyte/macrophage or T-cell infiltration. Of note, these and other early studies in animal models and human kidney samples focused primarily on expression patterns and associations of the CX3CL1-CX3CR1 axis in acute settings. Although clearly of potential mechanistic importance in the intense inflammation associated with acute kidney diseases, insights into the expression and functional significance of CX3CL1/CX3CR1 signalling in CKD was lacking. For the remainder of this article, we focus on subsequent evidence that CX3CL1 interaction with CX3CR1 is of distinct pathophysiological importance to the progression of fibrosis and impaired renal function in CKD.

## The Role of the CX3CL1-CX3CR1 Axis in Chronic Kidney Disease: Evidence From Animal Studies

Animal models have indicated that the CX3CL1-CX3CR1 axis is important in the progression of chronic renal diseases such as diabetic nephropathy (DN). Kikuchi et al. (42) showed, in rats with streptozocin-induced DN, that mRNA expression of CX3CL1 and CX3CR1 was increased four and eight weeks after induction of diabetes. Immunohistochemistry demonstrated that endothelial expression of CX3CL1 and number of CX3CR1 positive cells within the glomeruli were increased. Treatment with the ACE-inhibitor temocapril reduced intra-renal mRNA and protein levels of CX3CL1 and CX3CR1 eight weeks after induction of diabetes. Subsequently, micro-dissected glomeruli from normal rat kidneys were shown to upregulate CX3CL1 expression following *in-vitro* exposure to high concentrations of glucose, TNF-α or advanced glycation end-products (AGEs). These data demonstrate that CX3CL1 expression is induced in DN and may be modulated by therapeutic strategies already in use (43). The streptozocin-induced DN model was used in CX3CR1 knockout mice by Song et al. to further elucidate the role of CX3CL1 signalling in the development of DN (44). Renal fibrosis was induced in wild-type diabetic mice but was attenuated in CX3CR1-deficient animals. The streptozocin-treated knockout mice also had reduced mRNA expression of TGF-β, fibronectin and collagen-related genes and reduced macrophage accumulation within kidney tissue compared to their wild-type counterparts. *In vitro* work examined the role of CX3CL1 signalling in mesangial cells under diabetic conditions. In response to high glucose concentrations, mesangial cells increased expression of mRNA and protein for CX3CL1 and CX3CR1 and released greater amounts of CX3CL1 into the culture medium. Treatment of mesangial cells with CX3CL1 or with other diabetes-related stimuli also induced higher expression of the pro-fibrotic molecules TGF-β, collagen 4α1 and fibronectin at both mRNA and protein levels. Furthermore, small interfering (si)-RNA-mediated reduction of CX3CL1 expression attenuated these changes. These data highlight that CX3CL1 signalling *via* CX3CR1 contributes to macrophage infiltration and renal fibrosis in mouse models of DN and that mesangial cells both produce and respond to CX3CL1. Such findings related to mesangial cells in DN are consistent with reports in an acute glomerulonephritis model, indicating that CX3CL1, produced by mesangial cells plays a role in monocyte recruitment to the kidney (45) and in *in vitro* culture systems demonstrating that murine monocytes adhere to mesangial cells in a CX3CL1-CX3CR1 dependent fashion (46).

The importance of the CX3CL1-CX3CR1 axis in renal fibrosis following acute kidney injury has been studied by Furuichi et al. using an ischemia reperfusion injury (IRI) model. In this study, IRI resulted in increased expression of CX3CL1 protein in the kidney but did not demonstrate a corresponding change in mRNA levels. Furthermore, the location of CX3CL1 expression within the kidneys changed, being limited to endothelial cells in sham-operated animals while also detected on tubular epithelial cells and in association with infiltrating cells following IRI. Renal expression of CX3CR1 was increased at both protein and mRNA levels following IRI with protein expression mostly observed in association with infiltrating cells. The lack of correlation between mRNA and protein expression may simply reflect a technical limitation given the correlation between mRNA and protein level expression described by other authors. The severity of acute tubular necrosis following IRI did not differ between wild type (WT) and CX3CR1^-/-^ mice. However, collagen deposition was reduced in the CX3CR1^-/-^ animals, as was intra-renal macrophage infiltration while lymphocyte and neutrophil numbers were unaffected. Overall, this work was consistent with a role for CX3CR1-dependent macrophage-mediated fibrosis following IRI. To account for the possibility of developmental differences in macrophage lineages in CX3CR1^-/-^ mice, Furuichi et al. also used pharmacological blockade by administering a CX3CR1-neutralising antibody following IRI. Animals treated with the blocking antibody had significantly reduced post-IRI renal fibrosis compared to controls (47). Overall these data suggested that the CX3CL1-CX3CR1 axis was of mechanistic importance in the development of renal fibrosis and a potential for it to be targeted therapeutically.

Oh et al. also demonstrated increased CX3CL1 expression after IRI, using immunoblotting and immunohistochemistry. In this study, blockade of CX3CR1 was associated with protection against acute renal impairment, as assessed by histology and blood urea and creatinine levels, and with reduced macrophage infiltration (48). Another mouse model of IRI demonstrated that CX3CR1 deficiency is associated with protection against renal impairment after IRI and reduced infiltration of the renal parenchyma with monocyte-derived macrophages. Macrophage infiltration of the kidney was restored and protection from renal impairment attenuated after adoptive transfer of CX3CR1-competent blood monocytes (49). These results supported the conclusion that CX3CR1-signalling is required for monocyte/macrophage migration into the kidney and that CX3CR1-dependent migration has deleterious effects on renal function following IRI (49). Overall, these data from animal model studies are consistent with a mechanism by which intra-renal induction of CX3CL1 expression following IRI plays a key role in monocyte-derived macrophage infiltration of the renal parenchyma and in the subsequent severity of renal fibrosis. Whether this pathway is also an important, targetable mechanism of AKI progression to CKD in humans remains less well understood and merits more detailed investigation.

Other animal models of pro-fibrotic kidney injury have also provided compelling evidence of a role for CX3CL1 in mediating renal leucocyte infiltration and renal functional impairment. For example, in a mouse model of radiation-induced renal injury, increased CX3CL1 expression within glomeruli was observed at 30 and 40 weeks after irradiation (50) and correlated with areas of leucocyte infiltration on serial section analysis. Similarly, in a mouse model of hypertension, the combination of deoxycorticosterone and high salt diet following unilateral nephrectomy resulted in elevated mRNA for both CX3CL1 and CX3CR1 within the kidneys. In this study, CX3CL1 was chiefly expressed by PTEC, peritubular capillaries and vascular endothelial cells. Treated animals exhibited F4/80^+^ macrophage infiltration of the kidneys with interstitial fibrosis and these changes, along with intra-renal TGFβ1 level, were ameliorated in CX3CR1-deficient mice (51). Mice with cisplatin-induced renal impairment have also been reported to have increased CX3CL1 expression within the kidneys. However, in this model of acute kidney injury, neither treatment with an anti-CX3CR1 neutralising antibody nor genetic CX3CR1 deficiency reduced the peak creatinine level, peak blood urea nitrogen level or macrophage infiltration of the kidney after injury (52). In a rat model of renovascular hypertension, serum CX3CL1 levels are increased in hypertensive rats, with or without concomitant *Trypanosoma cruzi* infection (53). High fructose diet-induced kidney injury is also ameliorated in CX3CR1-deficient mice *via* a reduction in NF-kB signalling (54).

Utilising the mouse unilateral ureteric obstruction (UUO) model, Peng et al. investigated the role of CX3CL1 in recruitment of different monocyte subsets into the obstructed kidney (55). This model demonstrated that both Ly6C^hi^/CX3CR1^lo^ (classical) and Ly6C^lo^/CX3CR1^hi^ (nonclassical) monocytes are recruited to the kidney after obstruction, albeit within different time-frames. The authors reported that, while Ly6C^hi^ monocytes are the major producers of the proinflammatory cytokines TNF-α and IL-1β, Ly6C^lo^ monocytes produce more of the pro-fibrotic cytokines TGF-β and platelet-derived growth factor (PDGF). Similar to other reported models, renal fibrosis was attenuated in CX3CR1-deficient mice after UUO. Further analyses demonstrated a specific lack of nonclassical monocyte accumulation within the obstructed kidneys of CX3CR1-deficient mice. Interestingly, this deficit was due to increased apoptosis rather than impaired recruitment, in keeping with the role of CX3CR1 signalling in cell survival outlined (18). Several of the studies described heretofore have indicated that macrophage accumulation is promoted *via* the CX3CR1-CX3CL1 axis. Of relevance to this literature, recent work has shown that the CX3CL1-CX3CR1 axis mediates blood monocyte infiltration of the colon and, thus, contributes to macrophage accumulation and tissue damage (56). More specifically, it was shown that CX3CL1 mediates crawling of monocytes on the colonic veins and that blockade of CX3CR1 prevents monocyte crawling and macrophage accumulation within the colon. Similar mechanisms may be responsible for macrophage accumulation in kidney injury.

To better address the potential relevance of CX3CL1-mediated signalling in CKD, Koziolek et al. (57) used a mouse folic acid nephropathy (FAN) model. Animals treated with a single high-dose intraperitoneal injection of folic acid consistently developed progressive tubulointerstitial fibrosis with renal functional impairment and proteinuria. Subsequent analyses of FAN kidneys demonstrated upregulation of CX3CL1 and CX3CR1 mRNA and protein for more than a hundred days after treatment. Compared to control kidneys, the location of CX3CL1 expression changed from limited expression on endothelial cells to expression on glomerular and peritubular capillaries, mesangial cells, tubular cells and cells within the interstitium. Immunohistochemistry also demonstrated increased expression of collagen-1, CD3^+^ (T-cell) infiltrates and S100A4, a protein expressed by fibroblasts and associated with epithelial-to-mesenchymal transition (57). The intensity of CX3CL1 expression correlated with the degree of tubulointerstitial fibrosis. Further work utilised *in vitro* analyses of murine renal tubular epithelial cell lines and demonstrated that these cells were capable of upregulating CX3CL1 expression and demonstrated increased chemotactic potency for peripheral blood mononuclear cells after stimulation with cytokines such as TNF-α, IL-1β or TGF-β (57). This animal study indicated that many of the functional effects of CX3CL1 demonstrated in more acute models of renal disease are also relevant to CKD. Finally, treatment of human renal fibroblast cell lines with CX3CL1 was shown to further upregulate CX3CL1 expression and to result in increased cell numbers, due to both increased proliferation and increased survival (57).

In contrast to the studies summarized above, work by Engel et al. has raised questions about the role of CX3CR1-CX3CL1 signalling in promoting renal fibrosis, instead providing experimental evidence that CX3CR1 deficiency can result in accelerated renal fibrosis under some circumstances (58). In this study, renal fibrosis following UUO was increased in CX3CR1-deficient mice compared to their wild type counterparts. CX3CR- deficient mice had a slightly decreased total number of mononuclear phagocytes after UUO but further analyses demonstrated a relative increase in blood monocyte-derived macrophages with a decrease in resident macrophages and dendritic cells. TGF-β producing macrophages were also increased in the CX3CR1-deficient mice, suggesting a mechanism for increased fibrogenesis. Finally, this work indicated that increased macrophage proliferation within the kidney occurred in CX3CR1-deficient mice. Overall, the authors proposed that lack of the CX3CR1 receptor allows macrophage proliferation within the renal parenchyma after injury, thus driving renal fibrosis (58). More recently, Ahadazadeh et al. examined the role of CX3CR1 in angiotensin-induced hypertension in mice. Greater albuminuria, more severe glomerular injury and increased renal fibrosis were seen in the CX3CR1-deficient mice, although blood urea nitrogen did not differ from that of wild type mice. Alongside these differences, increased neutrophil and F4/80^+^ macrophage infiltration of the kidneys and reduced dendritic cell numbers were seen in the CX3CR1-deficient mice. Cardiac injury was also assessed and was not reduced by CX_3_CR1 deficiency. In a different disease scenario, it has also been demonstrated that CX3CR1-dependent mechanisms protect against acute kidney injury in sepsis (59). These studies, which indicate that there may be counter-regulatory roles for CX3CR1-expressing myeloid cells during pro-fibrotic renal inflammation, must be taken into consideration when evaluating the clinical potential of CX3CR1 blockade in human CKD from diverse causes. It should be noted, however, that they depend on unconditional, whole-animal knockout of CX3CR1. Thus, differences in embryonic development, such as altered seeding of the renal tissue yolk sac-derived macrophages, may have impacted on the study findings. In order to better determine the potential for CX3CR1 targeting studies, incorporating a pharmacological blockade strategy at the time of renal injury or an inducible CX3CR1 knockout model should be utilised wherever possible.

## The Role of the CX3CL1-CX3CR1 Axis in Chronic Kidney Disease: Evidence From Studies of Human Tissue and Cells

In 2007, Koziolek et al. studied CX3CR1 expression within the renal parenchyma of patients with >40% interstitial fibrosis associated with only minor cellular infiltration compared to normal renal parenchyma obtained at the time of tumour nephrectomy. In normal kidneys, CX3CR1 expression was absent or minimal but, in fibrotic kidneys CX3CR1^+^ cells were identified within both scarred glomeruli and the tubulointerstitial compartment. Serial sectioning indicated that some of the CX3CR1^+^ cells were CD68^+^ monocyte/macrophages or CD3^+^ T-cells but not all cells co-stained with these markers and double immunofluorescence studies indicated that, in advanced renal fibrosis, tubular epithelial cells, myofibroblasts (α-SMA^+^) and dendritic cells (CD11c^+^) also express CX3CR1. In keeping with this, human renal fibroblast cell lines also express CX3CR1 and migrate towards a CX3CL1 gradient suggesting a functional role for the CX3CL1- CX3CR1 in promoting renal fibrogenesis through fibroblast recruitment (60). Wang et al. demonstrated that exposure to AGEs increases human renal mesangial cell expression of CX3CL1. Production of matrix metalloproteinase two (MMP-2), an enzyme required for extracellular matrix degradation is inhibited in mesangial cells by AGEs and in a monocytic cell line by CX3CL1. Thus, extracellular matrix degradation may be reduced in diabetes due, in part, to increased CX3CL1 expression and this may contribute to renal extracellular matrix deposition and progression of renal impairment in DN (61).

In a different clinical setting, Yoshimoto et al. reported glomerular CX3CL1 expression and CD16^+^ monocyte infiltration in biopsies from people with acute lupus nephritis (62). Similar observations were subsequently reported in a mouse model of lupus nephritis. Of note, human CD16^+^ monocytes are now conventionally subdivided on the basis of CD14 expression into intermediate and nonclassical subsets (63) and have been shown to circulate in higher numbers in CKD, atherosclerosis and multiple other pro-inflammatory conditions (64, 65). Despite the demonstrated expansion of these populations in CKD and their association with CKD progression (64), the extent to which they infiltrate the renal parenchyma and contribute to renal inflammation and fibrosis during CKD is incompletely understood (66). Further investigation of the role of CX3CL1 in mediating inflammatory monocyte subset activities in human CKD will be of value in elucidating these issues.

In addition to its reported role in renal monocyte/macrophage recruitment and activation, data also exist to indicate that CX3CL1-CX3CR1 signalling is important for the recruitment and retention of dendritic cells (DCs) and for their pro-fibrotic effects in human kidneys. For example, Kassianos et al. used flow cytometric analysis of leukocytes within human fibrotic and non-fibrotic kidneys to demonstrate that CD1c^+^ DCs express CX3CR1 and are important producers of TGF-β within fibrotic kidneys. Immunohistochemical analyses of the same kidneys demonstrated upregulation of CX3CL1 on PTEC and co-localisation of CD1c^+^ DCs with the CX3CL1-expressing tubular cells (67). In vitro analyses confirmed that PTEC-derived CX3CL1 is capable of mediating chemotaxis and adhesion of DCs. Overall, this study provides strong evidence that upregulation of CX3CL1 by PTEC is a mechanism by which pro-fibrogenic DCs are recruited and retained in the renal parenchyma in CKD.

Of potential relevance to these tissue-based observations, a number of groups have investigated the association between single nucleotide polymorphisms (SNPs) within the CX3CR1 gene and the presence of CKD or end stage kidney failure (ESKF). One such polymorphism is V249I. The frequency of the I allele and the II genotype were found to be increased in ESKF by two groups (68, 69). In contrast, Yadav et al. reported that the II genotype was more common in controls (70) without significantly different allelic frequency of the I allele. Variations in the frequency of T280M CX3CR1 polymorphisms have also been observed in CKD. Interestingly, these same polymorphisms may influence the risk of cancer (71) and of delayed graft function (72) after renal transplantation.

## Atherosclerosis in Chronic Kidney Disease: Contribution of Systemic Dysregulation of the CX3CL1- CX3CR1 Axis

It is well established that atherosclerosis is accelerated and cardiovascular morbidity and mortality increased among people with CKD (73, 74). One contributing factor to the burden of cardiovascular disease in CKD is a chronic micro-inflammatory state which may include systemic activation of the CX3CL1- CX3CR1 axis. Leucocyte recruitment into the blood vessel wall is required for the development of atherosclerotic plaques and CX3CL1-CX3CR1-mediated signalling has been shown to be an important mechanism in this process (75–77). This has prompted several groups to address the role of the CX3CL1-CX3CR1 axis in atherosclerosis acceleration in CKD.

In the first place, it has been reported that serum CX3CL1 concentrations are higher in people with CKD compared to age-matched controls (70, 78, 79) and correlate with eGFR (80). Additionally, serum CX3CL1 concentration been shown to be predictive of all-cause mortality and myocardial infarction in CKD cohorts (80, 81). Such observations have also been made in non-CKD populations (82, 83). Expression of CX3CR1 by circulating leukocytes may also be altered in CKD or ESKF. Blood monocytes were reported to have higher CX3CR1 expression in dialysis patients (84), although it is unclear from the data reported whether this was caused by upregulation of CX3CR1 expression by the whole monocyte population or by an increase in the proportion of CX3CR1^hi^ intermediate and nonclassical monocytes. Increased CX3CR1 expression has been found in the arterial walls of people with CKD undergoing renal transplantation (80). The proportion of CX3CR1 expressing T-cells also increases in ESKF and CKD, largely due to an expansion of CX3CR1^hi^/CD4^+^/CD28^-^ T-cells, the proportion of which is associated with intima-media thickness of the common carotid artery (79). Overall, these data favour a conclusion that CX3CL1-CX3CR1 signalling is increased in CKD. From a mechanistic perspective, one study indicated that *in vitro* exposure to the uremic toxin p-cresol (85), reduced trans-endothelial leukocyte migration and endothelial cell production of CX3CL1. However, high p-cresol concentrations were used in this study with potential implications for physiological relevance.

Importantly, animal and human data have also suggested a specific role for CX3CR1 expression by T-cells in CKD-associated acceleration of atherosclerosis (86). In a mouse model of CKD, atherosclerosis was attenuated in CX3CR1-deficient animals. Although macrophage infiltration of atherosclerotic lesions was reduced, reconstitution with CX3CR1^+^ monocytes did not restore the deficit. Instead, the authors observed that CX3CR1 deficiency resulted in reduced T helper 17 (T_H_17) cell polarization within atherosclerotic plaques and that this was necessary for the development of CKD-driven atherosclerosis. Adoptive transfer of CX3CR1^+^ T-cells restored atherosclerosis progression in mice with renal impairment, suggesting a specific contribution of CX3CR1^+^ T-cells to atherosclerosis in setting. This is in keeping with the study of Yadav et al. which an association between intima-medial thickening of the carotid artery and CX3CR1^+^/CD4^+^ T-cells in patients with CKD (79). Kim et al. examined upregulation of CX3CL1 by endothelial cells and the interactions between blood monocytes, endothelial cells and CD4^+^/CD28^-^ T-cells under ESKF-like *in vitro* conditions (87). Exposure of cultured endothelial cells to conditioned medium from monocytes treated with the uremic toxin indoxyl sulfate or directly to recombinant TNF-α resulted in upregulation of cell surface CX3CL1. In contrast, exposure to indoxyl sulphate itself did not increase endothelial CX3CL1. Treatment of CD4^+^ T-cells with TNF−α increased the proportion of CD4^+^CD28^-^CX3CR1^+^ T-cells – a subpopulation that is increased in ESKF – and co-culture of endothelial cells with CD4^+^CD28^-^CX3CR1^+^ T-cells resulted in increased endothelial apoptosis. The authors propose that chronic uraemia leads to increased monocyte production of TNF-α which induces endothelial CX3CL1 expression and accumulation of CD4^+^ CD28^-^ CX3CR1^+^ T-cells. This T-cell population may then bind to CX3CL1-expressing endothelial cells expressing CX3CL1 and compromise endothelial integrity (87).

Activation of the renin-angiotensin system and systemic angiotensinaemia may also contribute to CX3CL1-CX3CR1 dysregulation in CKD. Li et al. recently reported that mice with renal impairment have elevated expression of CX3CL1 and CX3CR1 in the aortic wall and that these levels were reduced by treatment with losartan. *In vitro* work using vascular smooth muscle (MOVAS) cells also showed that treatment with angiotensin increased CX3CR1 expression and that this was reduced by concomitant treatment with losartan. The induced CX3CR1 was functional, leading to increased migration of angiotensin-treated MOVAS cells compared to untreated cells and to cells treated with angiotensin + losartan (88).

## Progress Toward Therapeutic Modulation of CX3CL1/CX3CR1 Axis in CKD

Modulation of fractalkine expression by existing medications or investigational medical products (IMP) has been reported and may be a mechanism by which they contribute to renal protection in CKD. This was highlighted in the animal studies above regarding RAS blockade (42). Mesenchymal stromal cells (MSCs) and MSC-derived microcellular vesicles (MSC-MVs) are recently developed advanced medical therapies with ongoing clinical trials for multiple medical conditions, including chronic kidney disease (89). A pre-clinical study investigated the effect of human umbilical cord-derived MSC-MVs on renal inflammation and impairment in a rat renal IRI model. Rats treated with MSC-MVs following IRI were protected from renal fibrosis and renal functional impairment two weeks after injury. Macrophage infiltration of the renal tissue and CX3CL1 expression within the kidney were also reduced in the MSC-MV treated group. *In vitro*, endothelial cells subjected to hypoxia also demonstrated increased CX3CL1 expression, which was reduced by MSC-MV treatment. MSC-MVs were also found to contain six micro-RNAs matched to CX3CL1 mRNA. Although not conclusive, this study suggests that modulation of CX3CL1 expression may be a mechanism by which MSCs or MSC-MVs exert therapeutic effects (90). Studies of MSCs in animal models of other diseases, including myocarditis and amyotrophic lateral sclerosis have pointed to an increase in CX3CL1 in MSC treated animals, although with an overall beneficial effect of MSC treatment (91, 92). These discrepancies could be the result of tissue specific effects, particularly in the central nervous system where the pro-survival effects of CX3CL1 may be neuroprotective under certain circumstances (93). Another investigational medical product M4PTB, a histone de-acetylase inhibitor, has been shown to decrease CX3CL1 expression and fibrosis in a mouse model of ischemia renal IRI (94)

One of the first reports of a strategy to develop a therapeutic antagonist of CX3CR1 was made in 2009 by Dorgham et al. (95). A CX3CR1 antagonist was developed using PCR mutagenesis and phage selection. The resulting antagonist, termed ‘F1’, was capable of inhibiting CX3CL1-induced calcium signalling and chemotaxis of both a HEK293 cell line and primary human leukocytes. The antagonist also inhibited adhesion of CX3CR1^+^ cell lines to CX3CL1 *in vitro*. Furthermore, the authors demonstrated that *in vivo* CX3CR1 antagonism with F1 led to reduced leukocyte recruitment in a mouse model of thioglycolate induced peritonitis. Neutralising antibodies are commonly used *in vitro* or *in vivo* to establish the pathogenic significance of a biological pathway and several experimental studies described in previous sections have utilized anti-CX3CR1 antibodies (35, 47, 48, 52). In the last two decades, monoclonal antibodies targeting pro-inflammatory chemokines or receptors have been a significant advance in treatment of numerous medical conditions, including malignancies and autoimmune disease. The potential for therapeutic targeting of the CX3CL1-CX3CR1 axis in inflammatory or fibrogenic diseases has been noted (96, 97). Toward this goal, the development of a variable domain of camelid, heavy chain only (VHH) antibody targeting CX3CR1 has recently been described in detail (98). A 30mg/kg dose of this “nanobody”, named BI665088, which was developed by Ablynx™ in collaboration with Boehringer Ingelheim™ was shown to inhibit atherosclerosis development in mice with knock-in human CX3CR1 expression. A press release in 2016 reported planned initiation of a Phase I trial of an anti-CX3CR1 nanonbody in patients with CKD but further details have not been published to date (99). An anti-CX3CL1 monoclonal antibody has also been developed (100) and data from early stage clinical trials suggests possible benefit in patients with rheumatoid arthritis (101).

Small molecule inhibitors of CX3CR1 also have therapeutic potential. The pharmaceutical company, Astra Zeneca, reported the development of orally available small molecule inhibitors of CX3CR1. These were developed by systematically varying compounds capable of binding other chemokine receptors and selecting those with high CX3CR1 binding capacity (102). One of the identified proteins, named AZD8797, was shown by Cederblad et al. to function as a non-competitive allosteric modulator of CX3CR1, capable of preventing both CX3CL1-CX3CR1-mediated adhesion and downstream G-protein mediated signalling pathways (103). Animal studies indicated that AZD8797 may be effective in treating multiple sclerosis (104, 105). E6130, a pyrrolidin-3-ylacetic acid derivative with CX3CR1 modulating capacity was patented by Eisai Co. in 2013 and subsequently published data demonstrated that this compound downregulates CX3CR1 expression on NK cells and prevents CX3CL1-mediated chemotaxis (106). Moreover, this compound reduces the severity of a murine model of inflammatory bowel disease. A further small molecule inhibitor of CX3CR1, termed JMS 17-2 has been developed and used in animal studies regarding the potential for therapeutic targeting of this axis to prevent cancer metastasis (107). Most of the therapies developed to date, summarised in **Table 2**, focus on blockade of the CX3CR1 receptor, either through direct binding of the active site or through allosteric modulation. Targeting of the downstream signalling pathways, as illustrated in **Figure 1**, may also be of therapeutic value, albeit with less specificity.

**Table 2:** Pharmacological agents targeting the CX3CL1-CX3CR1 axis currently reported to be in development or clinical trials and disease of interest.

Product Name	Company/Institution	Class	Animal Models Reported	Human Trials In Progress/Planned
**F1**	INSERM	Modified CX3CR1 Ligand	Periodontitis	N/A
**BI665088**	Boehringer-Ingelheim/ Ablynx	VHH Antibody to CX3CR1	Atherosclerosis	Chronic Kidney Disease
**E6011**	Eisai Co	Humanised monoclonal Antibody	Pharmacokinetics in cynomolgus monkeys reported	Rheumatoid Arthritis Inflammatory Bowel Disease
**AZD8797**	Astra Zeneca	Small molecule inhibitor	Spinal Cord Injury Multiple Sclerosis	N/A
**E6130**	Eisai Co	Small Molecule Inhibitor	Inflammatory Bowel Disease	N/A
**JMS 17-2**	Drexel University College of Medicine	Small Molecule Inhibitor	Breast Cancer Metastasis	N/A

IMP, Investigational Medical Product; N/A, Not applicable; VHH, Variable Domain of Camelid, Heavy Chain Only.

## Conclusions

**Figure 2:**
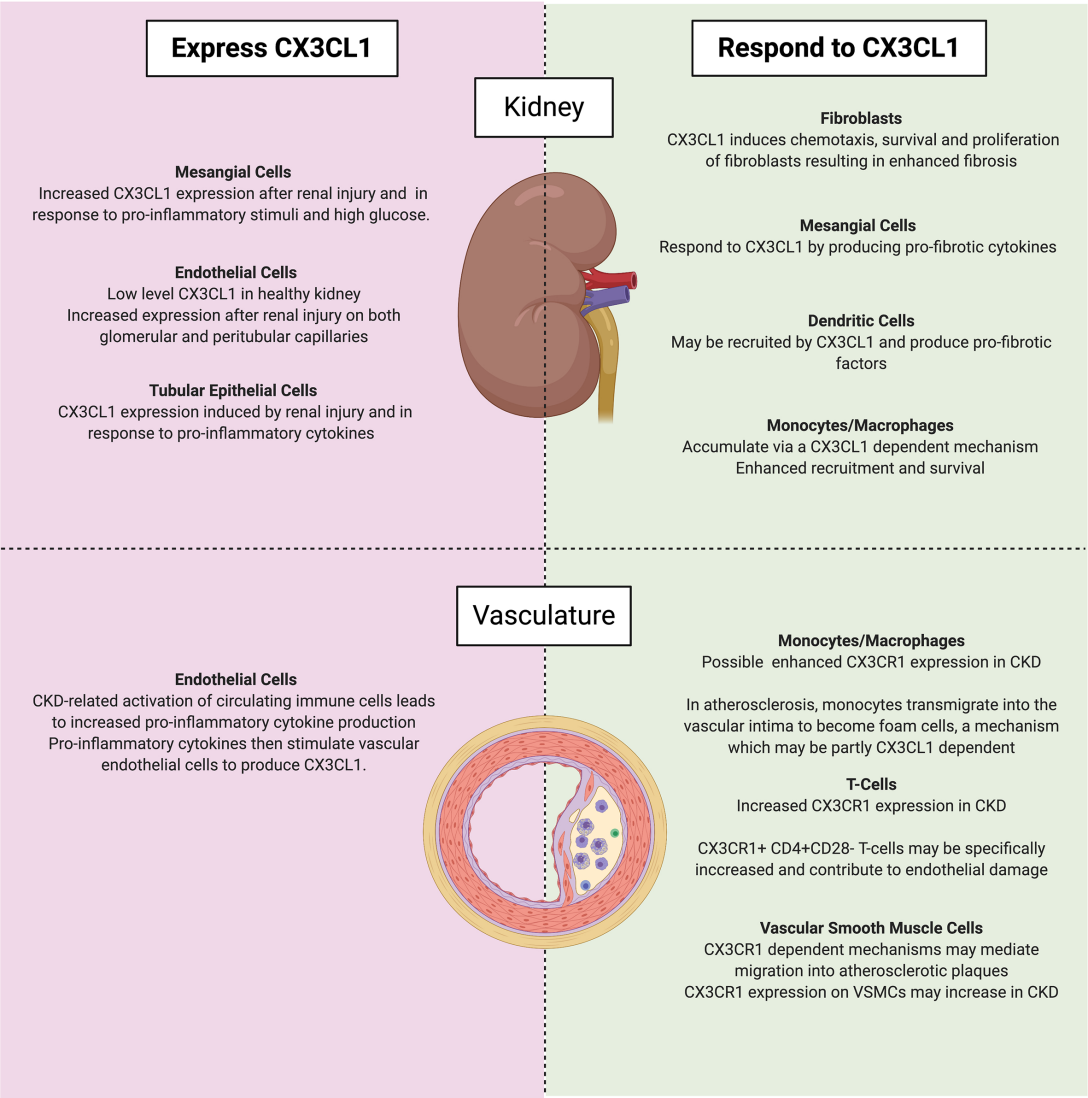
Cell types which either express (left) or respond to (right) CX3CL1 in CKD within the kidney (top) and vasculature (bottom). Of note, mesangial cells may both express and respond to CX3CL1. Created with BioRender.com.

More than 10% of the global population live with CKD (108). It is estimated that two million people worldwide have ESKF (109) and that more than one million deaths occur each year as a direct result of CKD (110). Despite this high burden, disease-modifying treatments for progressive CKD are lacking and new therapeutic strategies are needed. In this article we have highlighted a wealth of research from the past two decades that indicates a predominantly pathogenic role for the CX3CL1- CX3CR1 axis during both acute and chronic renal diseases. The CX3CL1-CX3CR1 axis is an attractive therapeutic target because it consists of a unique ligand/receptor pair which do not interact with other partners, limiting the potential for off-target effects. Multiple animal models have demonstrated that renal injury is associated with upregulation of CX3CL1 expression by multiple cell types within the kidney. This pathway is of particular importance in the recruitment of innate immune cells into the kidney tissue and to their subsequent pro-inflammatory and pro-fibrotic activities as illustrated by the role of CX3CL1-CX3CR1 in macrophage accumulation in the kidney. Furthermore, although not universally reported, several studies have indicated that either genetic or pharmacological targeting of the CX3CL1-CX3CR1 pathway attenuated renal fibrosis after injury. *In vitro* experiments have also demonstrated that exposure of renal tubular epithelial cells to CKD-related injurious stimuli such as high glucose, uremic toxins and pro-inflammatory cytokines may directly or indirectly induce increased CX3CL1 expression. In keeping with these observations, immunohistochemical studies of fibrotic human kidney specimens have demonstrated increased CX3CL1 expression and serial section analyses suggest an association between CX3CL1 expression and monocyte or T-cell recruitment. Interestingly, genetic polymorphisms in CX3CR1, may influence the risk of ESKF development, further suggesting a key role for this pathway in influencing progression of CKD in humans. Several studies suggest that dysregulation of the CX3CL1-CX3CR1 axis also occurs within the blood stream and vasculature of individuals with CKD and that this may contribute to the accelerated atherosclerosis typically seen in CKD. In **Figure 2**, we have summarised the cell types both within and outside of the kidney which produce or respond to CX3CL1. Finally, we have reviewed a number of recently developed small molecule inhibitors and monoclonal antibodies targeting the CX3CL1-CX3CR1 axis. We suggest that the time is ripe for further study of these agents in individuals with progressive fibrotic CKD.

## Author Contributions

SC wrote the first draft of the manuscript and revised subsequent drafts. MG reviewed and revised the manuscript. All authors contributed to the article and approved the submitted version.

## Funding

SC was supported by the Irish Clinical Academic Training (ICAT) Programme, supported by the Wellcome Trust and the Health Research Board (Grant Number 203930/B/16/Z), the Health Service Executive National Doctors Training and Planning and the Health and Social Care, Research and Development Division, Northern Ireland. MG was supported by grants from the European Commission [Horizon 2020 Collaborative Health Project NEPHSTROM (grant number 634086) and FP7 Collaborative Health Project VISICORT (grant number 602470)] and from Science Foundation Ireland [CÚRAM Research Centre (grant number 13/RC/2073)] and by the European Regional Development Fund.

## Conflict of Interest

The authors declare that the research was conducted in the absence of any commercial or financial relationships that could be construed as a potential conflict of interest.
